# Acupuncture for cancer symptoms: Clinical application and longitudinal impact a retrospective observational real-world data study

**DOI:** 10.1007/s00520-026-10372-z

**Published:** 2026-01-29

**Authors:** Wael Lasheen, Declan Walsh, Jonathan Polsky, Susan I. Yaguda, Beth York

**Affiliations:** 1 Department of Supportive Oncology, Levine Cancer Atrium Health, 1021 Morehead Medical Drive, Suite 70311, Charlotte, NC 28202 USA; 2 Department of Supportive Oncology, Levine Cancer Atrium Health, Charlotte, NC USA; 3 Hemby Family Endowed Chair in Supportive Oncology, Levine Cancer Atrium Health, Charlotte, NC USA; 4 RN Manager Integrative Oncology, Department of Supportive Oncology, Levine Cancer Atrium Health, Charlotte, NC USA; 5 Administrative Director, Department of Supportive Oncology, Levine Cancer Atrium Health, Charlotte, NC USA

**Keywords:** Acupuncture, Integrative medicine, Supportive care, Cancer, Symptoms, Disparities

## Abstract

**Purpose:**

Randomized controlled trials (RCTs) of acupuncture in oncology offer strong internal validity but limited generalizability. Real-world evidence is needed to assess feasibility, effectiveness, and adherence in routine practice.

**Methods:**

We conducted a retrospective analysis of real-world data from cancer patients receiving group Traditional Chinese acupuncture (ACP) in an outpatient oncology setting (2015–2022). Symptoms (anxiety, fatigue, hot flashes, neuropathy, pain, sleep) were rated on a 0–10 numeric scale before each session. Clinical improvement was defined as a ≥ 1-point decrease in severity.

**Results:**

A total of 2,239 patients underwent ACP (83% female; mean age 57 ± 12; 57% breast cancer). Common symptoms included pain (61%), sleep issues (50%), fatigue (45%), hot flashes (42%), anxiety (40%), and neuropathy (40%); 68% had ≥ 2 symptoms, median baseline severity 5–6. Females and younger patients had higher symptom burden. By session two, all symptoms improved statistically, with anxiety and hot flashes showing clinical improvement; by session three, all improvements were statistically and clinically significant. Benefits were similar across age and gender. Adherent patients (≥ 2 sessions) were older with higher symptom burden.

**Conclusion:**

Acupuncture appears effective for common cancer symptoms in real-world settings, with evidence suggesting that benefits are sustained between sessions and requiring multiple treatments for optimal effect. Both sexes and age groups experienced similar improvements, though utilization was lower among males and older adults. Expanding insurance coverage could improve access and reduce disparities. Adherence was high, and integration into outpatient oncology care proved feasible and sustainable. These findings warrant examination in RCTs.

**Supplementary Information:**

The online version contains supplementary material available at 10.1007/s00520-026-10372-z.

## Relevance of the manuscript to research, policies and/or programs

In the largest real-world study to date, we report acupuncture’s effectiveness in managing cancer-related symptoms. The findings suggest sustained benefits between treatments, the necessity for multiple sessions, and age and gender disparities, while identifying critical literature gaps to guide clinical practice. Furthermore, the study confirms the feasibility and sustainability of integrating complementary therapies into outpatient oncology, providing evidence to support expanded insurance coverage and informing research, policy development, and equitable program implementation.

## Introduction

Acupuncture (ACP), a form of Traditional Chinese Medicine (TCM), has experienced a steady increase in use within the United States [1]. Several factors have contributed to this rise: its integration into conventional clinical settings, shifting attitudes among patients and clinicians, coverage by some insurance plans, an expanding pool of ACP practitioners, and growing evidence supporting its efficacy [2, 3]. The theoretical foundation of ACP posits that the body’s vital energy, known as Qi, flows through specific pathways called meridians; disruptions or imbalances in this flow may lead to disease [4]. Stimulating acupuncture points are believed to restore Qi flow, thereby alleviating disease or symptoms [4].

Western interest in ACP surged following the National Institutes of Health’s 1997 consensus statement affirming sufficient evidence to support its use [5]. Since then, ACP’s role in supportive care and symptom management has expanded, driven by a growing body of randomized controlled trials (RCTs) [6]. This evidence has informed guidelines from organizations such as the American Society of Clinical Oncology-Society for Integrative Oncology and the National Comprehensive Cancer Network (NCCN), which endorse ACP for use during active cancer treatment and survivorship [1]. NCCN recommends ACP for managing hot flashes, cancer-related fatigue, and pain in cancer [7]. When performed by an experienced acupuncturist, complications are uncommon. The most common adverse events are bruising at the needle site and drowsiness, while serious risks, such as pneumothorax (occurring in two out of one-million cases), are rare [1]. ACP’s safety and lack of drug interactions, compared to pharmaceutical compounds, significantly enhances its appeal. In a survey of cancer patients (*n* = 457), 83% expressed interest in ACP and 63% perceived it as effective [8]. These findings underscore ACP’s growing acceptance in oncology care.

At Atrium Health Levine Cancer, the Department of Supportive Oncology established the Section of Integrative Medicine in 2013 to integrate complementary practices, such as acupuncture, healing touch, oncology massage, meditation, and tai chi, into conventional cancer care. A multidisciplinary team, comprising licensed practitioners, specialty-trained nurses, and physicians, delivers care in ambulatory settings. We believe this holistic, evidence-based approach promotes individualized patient care. In this study, we evaluate the longitudinal impact of serial ACP therapy on anxiety, fatigue, hot flashes, sensory neuropathy, pain, and sleep in cancer patients, alongside service utilization and ACP adherence in a conventional outpatient oncology practice.

## Material and methods

The aim of this study was to describe the impact of serial ACP therapy on cancer symptoms and patterns of use in real-world outpatient oncology practice. The primary objective was to evaluate the clinical response rate and improvement in severity of cancer-related symptoms, including anxiety, fatigue, hot flashes, sensory neuropathy, pain, and sleep, over serial ACP sessions. Secondary objectives included describing patient characteristics and comparing symptom improvement by age and gender, as well as determining adherence to ACP therapy. Outcome measures were defined as follows: symptom severity improvement was considered a ≥ 1-point improvement on a numeric rating scale between the first session and the second and third ACP sessions; clinical response rate was calculated as the percentage of patients with symptom improvement; symptom burden as the number of symptoms per patient; age groups were categorized as < 65 years and ≥ 65 years; gender was classified as female and male; and adherence was defined as the proportion of patients returning for a second ACP session.

### Design and study population

This study involved a retrospective analysis of a prospective cohort. Data analyzed was collected between 2015 and 2022, using a standardized intake form (Supplement 1), and Institutional Review Board (IRB00082670) approved. Written informed consent was waived. We adhered to the STRICTA (Standards for Reporting Interventions in Clinical Trials of Acupuncture) checklist to report our findings. Approximately half of the patients presented during cancer treatment, with the remainder seeking care at any point thereafter. Patients were either self-referred or physician referred. Before each treatment session, the clinician reviewed laboratory results, medical history, referral reason, and current medications.

Consecutive patients who completed symptom evaluation before treatment were included. For this analysis, eligibility criteria required patients to be over 18 years old, diagnosed with cancer, and did not receive other integrative service interventions. ACP exclusions included lack of consent, clinician concerns, immuno-compromised status, infectious disease, isolation precautions, psychosis or delusions, or skin conditions at needle insertion sites [9]. We operate on a fee-for-service model, with costs potentially offset by private insurance coverage or philanthropic support.

### Intervention

Our practice, rooted in TCM-based ACP, the most widely practiced approach in the USA, operates within a shared medical group setting, accommodating up to four patients simultaneously [10]. Patients are seated in adjustable reclining chairs, similar to those used in chemotherapy, encouraged to relax, complete intake forms, informed about the procedure and expectations, given the opportunity to ask questions, and informed consent obtained. Our acupuncturists are state-licensed, possess over 10 years of experience, and hold Master’s degree from an accredited acupuncture institute. Following skin sterilization, disposable sterile stainless steel filiform needles (Seirin, DBC, Acufast; gauge: 0.16 mm to 0.25 mm; length: 13 mm to 30 mm) are inserted one patient at a time. Approximately 18 needles are applied per session, with the number and insertion depth tailored to the patient’s symptom, previous response and treatment site. The acupuncturists target patient-reported symptoms, often addressing multiple issues concurrently. Additionally, Ashi points, ACP points located anywhere on the body, not necessarily along meridians, are occasionally used to relief musculoskeletal tension or tightness [11]. After insertion, needles remain in place for 30 to 45 min. Manual stimulation is applied at the practitioner’s discretion, while electrical stimulation is not utilized. We recommend one ACP session per week for four weeks, after which the treatment plan is individualized as needed. During ACP sessions, standard cancer treatments, medical and supportive care continued as usual.

### Assessment tools

A standardized intake form (Supplement 1) was completed before each ACP session. Patients identified their “most concerning symptom” then rated anxiety, fatigue, hot flashes, sensory neuropathy (numbness and tingling), pain, and sleep problems on an 11-point Numeric Rating severity Scale (NRS) ranging from 0–10 where 0 is “no symptom” and 10 “worst possible symptom”, except for sleep, which was anchored at 0 “restful” and 10 “not at all restful”. Anxiety, sensory neuropathy, and pain were assessed immediately following ACP sessions; however, only 30% to 50% of patients completed these evaluations because of time constraints. Due to incomplete immediate post-treatment evaluations for all symptoms and low completion rates, symptom severity reported immediately before the subsequent session was used to evaluate ACP response. The NRS for anxiety, fatigue, hot flashes, sensory neuropathy, pain, and sleep has been previously validated and/or applied in cancer studies [12–15]. Generally, an NRS of four or higher is considered moderate to severe and is clinically relevant, with a one-point change thereafter deemed clinically significant [16, 17]. In this study, the Minimal Clinically Important Difference (MCID) was defined as a change of ≥ 1 point on the 0–10 NRS. See the discussion section for further details on this threshold.

### Statistical analysis

Baseline variables for the entire study population are reported; thereafter, we present findings in symptom-specific subpopulations where a symptom was present (NRS ≥ 1) or identified as a symptom of concern. Standard descriptive statistics, including mean ± standard deviation (SD), median and interquartile range (Q1–Q3), and count (percent), were used to summarize patient characteristics, ACP utilization, and treatment outcomes. For continuous variables, we applied the Wilcoxon Rank Sum test with Monte Carlo estimates for exact testing to compare unpaired variables and the Wilcoxon Signed-Rank test for pairwise comparisons. For categorical variables, we employed the Chi-Squared test. Symptom burden was defined as the number of symptoms per patient. Clinical response rate was calculated as the percentage of patients achieving at least a one-point improvement on the NRS. Statistical tests were two-sided, with a p-value < 0.05 considered statistically significant. Analysis was conducted using SAS software (SAS® OnDemand for Academics, Cary, NC: SAS Institute Inc.).

## Results

### Patient characteristics and patterns of acupuncture utilizations

Between 2015 and 2022, we evaluated 2,239 consecutive patients (Table 1): 1,851 (83%) were female; the mean age ± SD was 57 ± 12; 1,276 (57%) had breast cancer; pain was the most concerning symptom, reported by 1355 (61%), while anxiety was the least concerning, reported by 833 (37%). At baseline, all symptoms were rated 5–6 in severity on the NRS (0–10). Most patients, 1524 (68%), presented with multiple symptoms (two or more), and 1867 (83%) completed at least two ACP sessions.

**Table 1:** Baseline Demographic Characteristics and Acupuncture Utilization

		Count (Percent) *N* = 2239 (100%)	
Age (Years)	Mean ± Standard Deviation	57 ± 12	
Gender	Female Male	1851 (83%) 388 (17%)	
Cancer Diagnosis	Breast Hematologic Gastro-Intestinal Gynecologic Lung Prostate Other	1276 (57%) 207 (9%) 192 (9%) 177 (8%) 82 (4%) 63 (3%) 229 (10%)	
Cancer Treatment (Past/Present)	Surgery Chemotherapy Radiotherapy	1614 (72%) 1379 (62%) 996 (44%)	
Symptoms Frequency at Basseline*	Pain Sleep Problems Fatigue Neuropathy Hot Flashes Anxiety Other	1355 (61%) 1076 (48%) 991 (44%) 927 (41%) 835 (37%) 833 (37%) 386 (17%)	
Symptoms Severity: on NRS at Baseline		**Mean** **(SD)**	**Median** **(Q1-Q3)**
	Anxiety (*N* = 827)	5 ± 3	5 (2–7)
	Fatigue (*N* = 986)	5 ± 3	5 (3–7)
	Hot Flashes (*N* = 834)	6 ± 3	6 (4–8)
	Neuropathy (*N* = 927)	5 ± 3	5 (3–8)
	Pain (*N* = 1355)	5 ± 2	5 (3–7)
	Sleep Problems (*N* = 1071)	6 ± 2	6 (4–8)
Number of Symptoms Per Patient	1 ≥ 2	32% 68%	
Number of Acupuncture Visits	1 ≥ 2 ≥ 3 ≥ 4	2239 (100%) 1867 (83%) 1579 (71%) 1255 (56%)	
Interval between Acupuncture Sessions (Days)		**Median (Q1-Q3)**	
	1st—2nd	7 (7–14)	
	2nd −3rd	7 (7–14)	
	3rd – 4th	7 (7–14)	

(Q1-Q3): Interquartile Range

(SD): standard Deviation

NRS: Numeric Rating Scale (0–10)

^*^Patient could present with multiple symptoms

### Change in symptom severity and clinical response rates

**Fig. 1:**
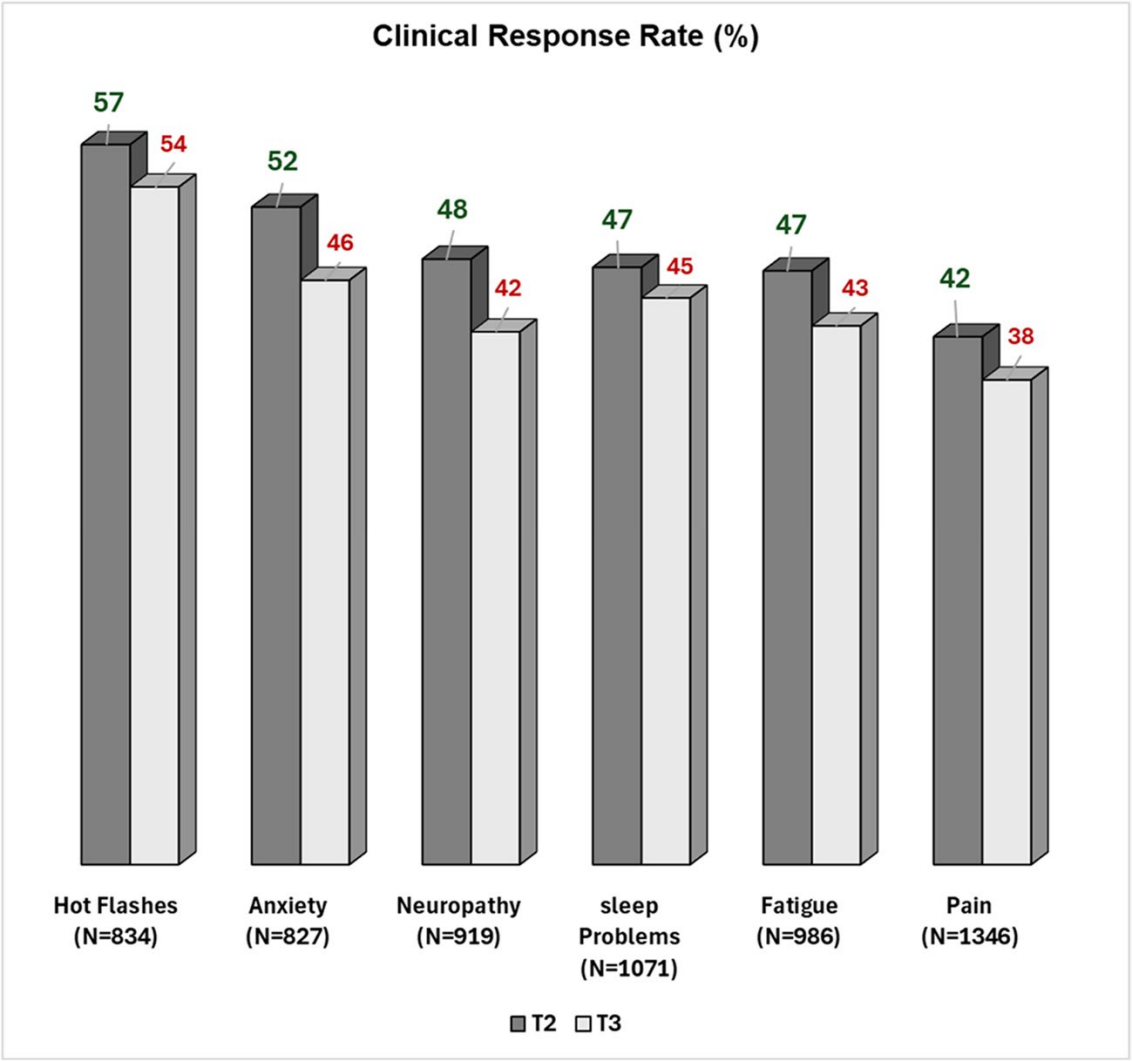
Clinical Response Rates^1^ for All Patients by Second (T2) ^2^ and Third (T3) Acupuncture Sessions^3^ Neuropathy = tingling and numbness.^1^Percentage of Patients with Clinical Symptom Severity improvement (≥ 1-points) on a severity numeric rating scale).^2^Those with only one acupuncture session counted as non-responders.^3^Those with only two acupuncture sessions counted as non-responders

**Fig. 2:**
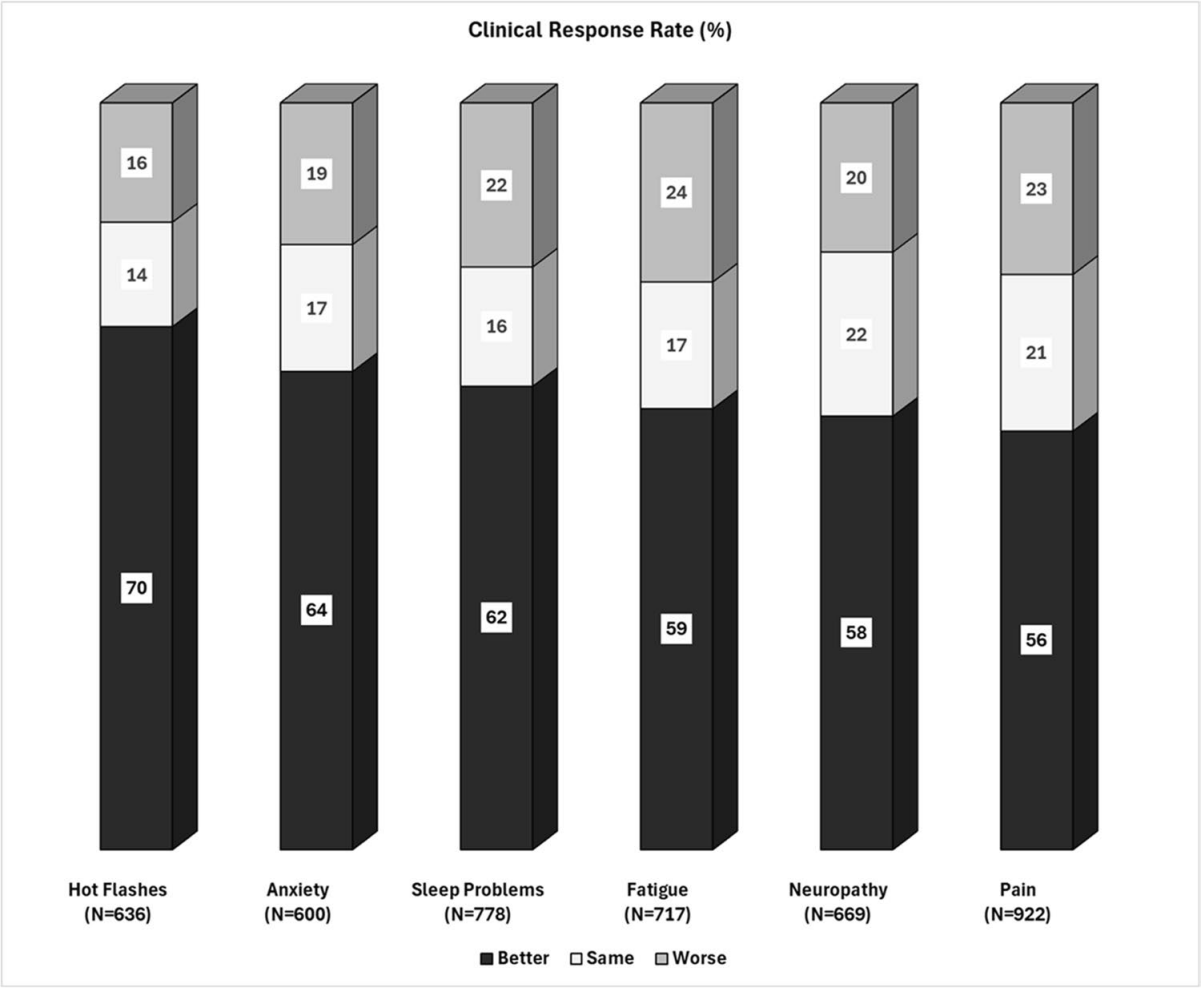
Clinical Response Rates* In Those Who Attended at Least Two Acupuncture Sessions or More. Neuropathy = tingling and numbness.^*^Percentage of Patients with Clinical improvement: 1-point or more on severity Numeric Rating Scale (0–10)

By the second ACP session, all symptoms showed statistically significant improvement. However, only anxiety and hot flashes also demonstrated clinically meaningful improvement (Table 2). By the third session, improvements were both statistically significant and clinically meaningful across all symptoms. Among all patients, those who did not return for a second ACP session were classified as non-responders, clinical response rates ranged from 57% for hot flashes to 42% for pain (Fig. 1). In those completing two ACP sessions or more (excluding those who did not return for further ACP), clinical response rates varied from 70% for hot flashes to 56% for pain (Fig. 2).

**Table 2:** Symptom Severity at Baseline (T1), Second (T2), and Third (T3) Acupuncture Sessions, and Mean Change from Baseline as Measured on a Severity Numeric Rating Scale (NRS: 0–10)

	Acupuncture Session	N	Median Severity NRS (Q1-Q3)	Mean Change ± Standard Deviation	P-Value*
Anxiety	**T1**	827	5 (2–7)		
	**T2**	**702**	**3 (1–5)**	**−1.2 ± 2.4**	< 0.0001
	**T3**	**600**	**3 (1–5)**	**−1.5 ± 2.5**	< 0.0001
Fatigue**	**T1**	986	5 (3–7)		
	**T2**	**840**	**4 (2–6)**	**−0.9 ± 2.5**	< 0.0001
	**T3**	**721**	**4 (2–6)**	**−1.1 ± 2.7**	< 0.0001
Hot Flashes	**T1**	834	6 (4–8)		
	**T2**	**730**	**4 (2–7)**	**−1.6 ± 2.8**	< 0.0001
	**T3**	**636**	**4 (1–7)**	**−2.0 ± 2.9**	< 0.0001
Neuropathy	**T1**	919	5 (3–8)		
	**T2**	**805**	**4 (2–6)**	**−0.9 ± 2.3**	< 0.0001
	**T3**	**674**	**4 (2–6)**	**−1.0 ± 2.4**	< 0.0001
Pain	**T1**	1346	5 (3–7)		
	**T2**	**1127**	**4 (2–6)**	**−0.7 ± 2.4**	< 0.0001
	**T3**	**929**	**3 (2–5)**	**−1.0 ± 2.5**	< 0.0001
Sleep Problems	**T1**	1071	6 (4–8)		
	**T2**	**914**	**5 (3–7)**	**0.9 ± 2.8**	< 0.0001
	**T3**	**781**	**4 (2–6)**	**−1.3 ± 2.8**	< 0.0001

Q1-Q3 interquartile range

^*^Wilcoxon Signed-Rank test for pairwise comparison

^**^Those who discontinued Acupuncture had more severe Fatigue (P-Value < 0.01) at baseline

### Acupuncture utilization by gender

Of the total cohort, 1,851 (83%) were female and 388 (17%) were male (Table 3). Females were significantly younger than males (mean age 56 ± 12 years vs. 61 ± 13) and had a higher symptom burden (3 ± 2 symptoms/patient vs. 2 ± 2). At baseline, all symptoms were more prevalent in females, except sensory neuropathy, which was more common in males, in this sample. Among females, sleep problems were most frequent, affecting 931 (50%), and pain was least frequent, affecting 732 (40%). Among males, sensory neuropathy was most prevalent, affecting 186 (48%), while hot flashes were least common, affecting 66 (17%). At baseline, anxiety, hot flashes, and sleep problems were more severe in females; sensory neuropathy was more severe in males; and there were no differences in pain severity. No gender differences were observed in clinical response rates to ACP.

**Table 3:** Patient Characteristics and Acupuncture Utilization Patterns by Gender

	Female *N* = 1851	Male *N* = 388	
Age			**P-Value**^**1**^
(Mean ± SD)	**56 ± 12**	**61 ± 13**	** < 0.0001**
Symptom Burden^3^			**P-Value**^**1**^
(Mean ± SD)	**2.9 ± 2**	**2.4 ± 2**	** < 0.0001**
Primary Cancer Site Breast Gynecologic Prostate Hematological Gastrointestinal Lung Other	**1274 (69%)** **177 (10%)** **0** **111 (6%)** **107 (6%)** **52 (3%)** **118 (6%)**	**2 (1%)** **0** **63 (16%)** **96 (25%)** **85 (22%)** **30 (8%)** **111 (29%)**	**P-Value**^**2**^ ** < 0.0001**
Cancer Treatment	**Count (%)**	**Count (%)**	**P-Value**^**2**^
(Past or Present)			
Chemotherapy	**1097 (59%)**	**282 (73%)**	** < 0.05**
Radiotherapy	**849 (46%)**	**147 (38%)**	** < 0.05**
Surgery	**1440 (78%)**	**174 (45%)**	** < 0.05**
Number of Acupuncture sessions			**P-Value**^**2**^
1	**1851 (100%)**	**388 (100%)**	**_**
≥ 2	**1546 (82%)**	**321 (83%)**	**NS**
≥ 3	**1314 (71%)**	**263 (68%)**	**NS**
≥ 4	**1056 (57%)**	**199 (51%)**	** < 0.05**
Symptom Frequency^4^	**Count (%)**	**Count (%)**	**P-Value**^**2**^
Anxiety	**732 (40%)**	**101 (26%)**	** < 0.05**
Fatigue	**841 (45%)**	**150 (39%)**	** < 0.05**
Hot Flashes	**769 (42%)**	**66 (17%)**	** < 0.05**
Neuropathy	**741 (40%)**	**185 (48%)**	** < 0.05**
Pain	**1130 (61%)**	**225 (58%)**	**NS**
Sleep Problems	**931 (50%)**	**143 (37%)**	** < 0.05**
Symptom Severity at Baseline^5^	**Median (Q1-Q3)**	**Median (Q1-Q3)**	**P-Value **^**1**^
Anxiety	**5 (3–7)**	**4 (2–6)**	** < 0.05**
Fatigue	**5 (3–7)**	**5 (3–7)**	**NS**
Hot Flashes	**6 (4–8)**	**5 (3–8)**	** < 0.05**
Neuropathy	**5 (3–7)**	**6 (4–8)**	** < 0.05**
Pain	**5 (3–6)**	**5 (3–7)**	**NS**
Sleep Problems	**6 (4–8)**	**5 (3–7)**	** < 0.05**
Clinical Response Rate^6^: T2-T1 Anxiety Fatigue Hot Flashes Neuropathy Pain Sleep Problems	**Count (%)** **384 (62%)** **404 (56%)** **445 (66%)** **357 (62%)** **462 (49%)** **451 (57%)**	**Count (%)** **47 (64%)** **60 (54%)** **31 (61%)** **84 (60%)** **101 (57%)** **56 (50%)**	**P-Value**^**2**^ **NS** **NS** **NS** **NS** **0.06** **NS**

Q1-Q3: interquartile range

SD: standard deviation

T1: Baseline

T2: Second Acupuncture session

^1^Wilcoxon Rank Sum with Monte Carlo estimates for the exact test

^2^Chi-Squared test

^3^Symptom Burden = Mean number of symptoms per patient

^4^Patients may present with multiple symptoms

^5^Symptom Severity measured on a Numeric Rating Scale (NRS: 0–10)

^6^An improvement of one or more points on a NRS by the second acupuncture session (T2);

### Acupuncture utilization by age group

Of the total sample, 1661 (74%) were under 65 years old, predominantly female, and diagnosed with breast cancer (Table 4). At baseline, younger patients had a higher symptom burden (2.9 ± 2 vs. 2.6 ± 2) compared to those 65 and older. In younger patients, pain was the most frequent symptom, affecting 994 (60%), and anxiety the least frequent, affecting 648 (39%). In older patients (≥ 65), pain was the most frequent, affecting 361 (62%), while hot flashes were the least frequent, affecting 131 (23%). At baseline, hot flashes and sleep problems were more severe in younger patients, whereas sensory neuropathy was more severe in older patients. Clinical response rates to ACP did not differ by age group.

**Table 4:** Patient Characteristics and Acupuncture Utilization by Age Group (< 65 years vs ≥ 65)

	Age < 65 *N* = 1661 (74%)	Age ≥ 65 *N* = 578 (26%)	
Female Gender	**Count (%)** **1435 (86%)**	**Count (%)** **416 (72%)**	**P-Value**^**1**^ ** < 0.05**
Symptom Burden*	**Mean ± SD** **2.9 ± 2**	**Mean ± SD** **2.6 ± 2**	**P-Value**^**2**^ ** < 0.05**
Cancer Diagnosis: Breast Gastrointestinal Gynecological Hematologic Lung Prostate Other	**Count (%)** **1029 (62%)** **130 (8%)** **118 (7%)** **129 (8%)** **48 (3%)** **24 (1%)** **173 (10%)**	**Count (%)** **247 (43%)** **62 (11%)** **59 (10%)** **78 (14%)** **34 (6%)** **39 (7%)** **56 (10%)**	**P-Value **^**1**^ ** < 0.05**
Cancer Treatment (Past/Present) Chemotherapy Surgery Radiotherapy	**Count (%)** **1031 (62%)** **1219 (73%)** **751 (45%)**	**Count (%)** **348 (60%)** **395 (68%)** **245 (42%)**	**P-Value **^**1**^ **NS** ** < 0.05** **NS**
Number of Visits per	**Count (%)**	**Count (%)**	**P-Value **^**1**^
Patient			
1	**1661 (100%)**	**578 (100%)**	**_**
≥ 2	**1368 (82%)**	**499 (86%)**	** < 0.05**
≥ 3	**1154 (69%)**	**425 (74%)**	** < 0.05**
≥ 4	**904 (46%)**	**351 (61%)**	** < 0.05**
Symptoms Frequency at Baseline** Pain Sleep Problems Fatigue Neuropathy Hot Flashes Anxiety	**Count (%)** **994 (60%)** **832 (50%)** **762 (46%)** **649 (39%)** **704 (42%)** **648 (39%)**	**Count (%)** **361 (62%)** **244 (42%)** **229 (40%)** **278 (48%)** **131 (23%)** **185 (32%)**	**P-Value **^**1**^ **ns** ** < 0.05** ** < 0.05** ** < 0.05** ** < 0.05** ** < 0.05**
Symptom Severity at	**Median (Q1-Q3)**	**Median (Q1-Q3)**	**P-Value**^**2**^
Baseline (NRS)			
Anxiety	**5 (3–7)**	**4 (2–7)**	**ns**
Fatigue	**5 (3–7)**	**5 (3–7)**	**ns**
Hot Flashes	**7 (4–8)**	**5 (3–8)**	** < 0.05**
Neuropathy	**5 (3–7)**	**6 (4–8)**	** < 0.05**
Pain	**4 (3–6)**	**5 (3–7)**	**ns**
Sleep Problems	**6 (4–8)**	**5 (3–8)**	** < 0.05**
Clinical Response	**Count (%)**	**Count (%)**	**P-Value**^**1**^
Rate***: T2-T1			
Anxiety	**335 (62%)**	**96 (63%)**	**ns**
Fatigue	**351 (55%)**	**113 (58%)**	**ns**
Hot Flashes	**397 (65%)**	**79 (68%)**	**ns**
Neuropathy	**312 (62%)**	**129 (59%)**	**ns**
Pain	**416 (51%)**	**147 (48%)**	**ns**
Sleep Problems	**401 (58%)**	**106 (50%)**	**ns**

**Q1-Q3: interquartile range**

**SD: standard deviation**

**T1: first acupuncture session**

**T2: second acupuncture session**

**NRS: Numeric Rating Scale (NRS: 0–10)**

^*^Symptom Burden = Mean number of symptoms per patient

^**^Patients may present with multiple symptoms

^***^An improvement of one or more points on a NRS by the second acupuncture session

^**1**^**Chi-Squared test**

^**2**^**Wilcoxon Rank Sum with Monte Carlo estimates**

### Acupuncture adherence

At baseline, patients adhering to ACP (defined as two or more sessions) were older, had a higher symptom burden, and reported more frequent hot flashes and sensory neuropathy. Those attending only one treatment session reported higher fatigue severity. No differences were observed by gender or primary cancer sites (Supplement 2).

## Discussion

In the largest longitudinal study of consecutive cancer patients undergoing multiple ACP treatments, utilizing real-world data (RWD), we observed improvements in cancer-related symptom severity with ACP. Symptom relief was sustained between treatment sessions. And response to ACP improved with repeated intervention. ACP was widely accepted, with 83% returning for a second session. Both females and males, as well as older and younger adults, experienced similar benefits. Clinical response rates were comparable to those of other interventions [18].

Randomized controlled trials remain the gold standard in medical research; however, ACP presents unique challenges, particularly with blinding and sham controls. Sham ACP involves skin stimulation, which can trigger emotional, hormonal, and symptomatic effects, complicating interpretation of outcomes [19]. Conversely, RWD studies are increasingly recognized by the medical community and the FDA for intervention evaluation and approval [20]. These studies are more cost-effective, faster, and simpler to conduct, and they often reveal evidence overlooked by RCTs. For example, RWD can assess effectiveness and adverse events in minority and older populations; groups typically underrepresented in RCTs, while providing insights into real-world impact, feasibility, tolerability, and adoption.

Defining MCID is a key challenge in ACP assessment. It is essential for evaluating an intervention benefit and may vary by cancer type, symptom domain and severity, and individual factors. In breakthrough cancer pain (*n* = 130), “adequate relief”, forgoing rescue medication, was linked to a 2-point or 33% pain score reduction [21]. In chronic non-cancer pain (*n* = 2,724), MCID varied by baseline severity: 1, 1.7, and 2.8 points for NRS scores of 4–5, 6–8, and 9–10 [22]. For advanced cancer, Mathias et al. (*n* = 1,564) identified a 2-point MCID for pain, while Hui et al. (*n* = 796) found 1 point sufficient for multiple symptoms [16, 23]. Some conflate “adequate relief (absent need for rescue medication)” with MCID, as medication needs offer an objective measure where outcomes are scarce. Clinically, both matter: MCID may guide titration, while lack of adequate relief signals treatment changes. We defined MCID as a ≥ 1-point reduction on the NRS, based on Hui et al.’s confirmation in multiple symptoms [16], the fact that a one-point change is an acceptable MCID for baseline pain ≤ 5 (half our patients) [22], and to account for assessment timing. Patients were evaluated at least one week after ACP, which may reflect waning treatment effects; those assessed immediately post-intervention showed a more marked improvement. Supplement 3; immediate post intervention changes in symptom severity.

We identified three comparable ACP studies in real-world settings. De Valois et al. (*n* = 300) reported a 15-year experience with auricular ACP for breast cancer survivors focusing solely on hot flashes [24]. Zayas et al. (*n* = 241), conducted a cross-sectional study among breast cancer survivors, assessing multiple symptoms via a mailed questionnaire; most participants recalled symptom improvement [25]. Lopez et al. (*n* = 375) described their experience at The University of Texas MD Anderson Cancer Center, in an outpatient population like ours, predominantly comprising women, and breast cancer diagnosis [15]. Like us, Lopez et al. noted a younger cohort but reported less severe symptoms at presentation, possibly due to our larger sample size. They also reported ACP effectiveness and recommended one or two sessions per week. In our experience, a once-weekly cadence is more practical and acceptable to patients. Although we did not record/report detailed intervention complications, we, like in other studies, observed bruising and slight pain in some, and none experienced severe adverse events.

Fewer men and older adults utilized ACP. Evidence shows that these groups are less likely to use supportive care and, when enrolled, often present with advanced disease [26, 27]. Reasons for gender disparity remain unclear; proposed factors include misconceptions about attention-seeking and masculinity, though recent literature critiques this as oversimplified “victim-blaming” [28, 29]. In addition, few RCTs examine supportive care in men and older adults. In a brief PubMed search (using the criteria “acupuncture” AND “cancer” in the title, filtered by “RCT”) we identified 37 RCTs in breast cancer but only two in prostate cancer (Supplement 4. PubMed Search). Although women report more prevalent and higher distress, distressed men have poorer outcomes and shorter survival across most cancers [30–32]. This is possibly due to questionnaires failing to capture male-specific experience and underestimating symptoms and quality-of-life impairment [33]. Significant knowledge gaps remain, limiting service access in the underserved groups we identified.

Acupuncture adoption faces barriers related to cost, individual, and organizational factors [34]. Most frequently cited challenges include ACP availability, insufficient knowledge of supporting evidence, and insurance constraints. Under fee-for-service models, cost-effectiveness analysis (CEA) is critical, yet no U.S.-based CEA in cancer was identified. A Canadian non-inferiority RCT, in cancer, found similar symptom improvement for group and individual ACP, with group sessions costing roughly half ($200 vs. $400) and showing a trend toward better Quality-Adjusted Life Years, QALYs [10, 35]. Interviews revealed that group ACP offered social support, while individual ACP participants prioritized privacy and one-on-one interaction [36]. In another report, the implementation of group ACP increased service utilization by 275% [37]. European and Asian studies in non-cancer populations reported comparable benefits, with initial costs offset by reduced resource use and QALY gains, though results varied by disease and symptom type [38].

We observed that ACP benefits accumulated over multiple treatments for some symptoms, such as pain and cancer-related fatigue reflecting a dose response relationship. For symptoms with more acute nature, such as anxiety and hot flashes, the therapeutic effect was more pronounced and immediate. This was observed in RCTs. An RCT conducted at Memorial Sloan Kettering Cancer Center compared ACP to usual care and reported that pain relief increased progressively over 12-weeks, with effects sustained for an additional 12-weeks post-treatment [39]. In contrast, an RCT by Shen et al. evaluating ACP for chemotherapy-induced nausea and vomiting reported immediate relief, but the effect quickly diminished after treatment cessation [40].

In the course of this work, we identified substantial gaps in the existing evidence base, gaps that extend beyond ACP overall effectiveness to the nuanced aspects of its implementation. Future research should address the following priorities:

1. Gender and Age Disparities.

Investigate the underlying determinants of disparities in ACP access across gender, age, and potentially race. More importantly, develop and evaluate targeted strategies to mitigate these inequities and tailor ACP interventions to promote equitable care delivery.

2. ACP Benefits in Diverse Populations.

Address persistent limitations in research funding and the inadequate inclusion of diverse populations in many U.S.-based studies. These shortcomings have resulted in critical knowledge gaps regarding the benefits of ACP among underserved groups.

3. Prescribing Practices and Dose–Response Relationship.

Validate observations of sustained ACP benefits between sessions and explore potential dose-response effects. Such evidence will guide prescribing practices and inform clinical trial design.

4. Economic Evaluation and Cost-Effectiveness.

Within the current fee-for-service reimbursement structure, rigorous cost-effectiveness analysis in oncology is urgently needed. These findings will facilitate broader adoption of ACP and support policy decisions regarding insurance coverage.

5. Minimal Clinically Important Difference Standardization.

Existing literature reports multiple MCID thresholds across symptoms without accounting for contextual variables such as care setting, disease type, timing of assessment, and intended purpose. Further research and consensus-building are required to establish standardized MCID criteria, thereby improving consistency in reporting, interpretation, and comparability of findings.

This study has several strengths and limitations. This was a retrospective study, not an RCT. The potential for bias and confounding cannot be overlooked, particularly the risk of selection bias due to self-referral, which may confound findings due to higher motivation or experience. Although multiple symptoms were represented, these findings may not be generalizable to all symptoms. Data were derived from an internal registry that was not originally designed for research purposes; consequently, several important clinical variables were not captured, limiting our ability to conduct more advanced analyses of improvement, adherence, or response predictors. Furthermore, this study reflects the experience of a single large urban cancer center, which may not be generalizable to other settings. It is noteworthy that patients often returned only when symptoms worsened and did not return after completing active treatment or when continuing therapy, including ACP, elsewhere. This pattern may have led to an underestimation of adherence and true response rates. In this study, we sought to bridge the gap between RCTs, which are conducted in highly controlled environments and offer strong internal validity but limited generalizability, and real-world clinical practice. We analyzed the largest cohort to date of consecutive cancer patients receiving multiple ACP sessions, in a real-world setting, and reported ACP effectiveness across multiple symptoms, like in RCTs, in a representative sample of individuals seeking supportive care.

Our findings have important clinical implications. We demonstrated that integrating ACP into conventional outpatient oncology settings is feasible. While clinical trials often employ multiple treatment sessions per week, our results suggest that a single weekly ACP session is both acceptable and easier to adhere to, without compromising treatment effectiveness. Second, the use of a group ACP model enabled delivery of a more cost-efficient service. Although some patients were referred by physicians, many independently sought complementary services, highlighting the growing demand for supportive care despite out-of-pocket costs. These observations underscore the need to tailor ACP approaches for underserved populations. Finally, our findings support the case for expanded insurance coverage for ACP services.

## Conclusions

This study aimed to bridge the gap between randomized clinical trials, which offer limited generalizability, and real-world clinical practice. Our findings suggest that acupuncture alleviates common cancer-related symptoms in real-world settings, like in randomized controlled trials. Improvement appears sustained between acupuncture sessions and multiple treatments are needed for optimal benefit. Both males and females, as well as younger and older adults, experienced comparable benefits; however, utilization remained lower among males and older adults. Treatment adherence was high, and integration of acupuncture into conventional outpatient oncology practice proved feasible and sustainable. These observations warrant examination in randomized controlled trials. Given it demonstrated effectiveness and feasibility, expanding insurance coverage for acupuncture could improve access and reduce disparities in supportive cancer care. Future research should focus on cost-effectiveness analyses, strategies to increase uptake among underserved populations, optimal prescribing patterns, and standardized outcome measures to guide clinical and policy decisions.

## Supplementary Information

Below is the link to the electronic supplementary material.

Supplementary file1 (DOCX 563 KB)

Supplementary file2 (DOCX 18 KB)

Supplementary file3 (DOCX 15 KB)

Supplementary file4 (DOCX 25 KB)

## Data Availability

The dataset is not publicly available due to patient privacy issues. The data may be made available by the corresponding author upon reasonable written request.

## Declarations

### Oral Presentation

Lasheen W, Polasky J, Walsh D, Yaguda S. Acupuncture for Cancer Symptoms: Clinical application and longitudinal impact. Society of Integrative Oncology (SOI); Santa Mesa, California; October 25-27th 2024.
