# Supplementary figures and images for: Acupuncture for cancer symptoms: Clinical application and longitudinal impact a retrospective observational real-world data study
Source: Support Care Cancer. 2026 Jan 29;34(2):145. doi: 10.1007/s00520-026-10372-z (PMC12855393; doi:10.1007/s00520-026-10372-z)

Online Resource 1. Acupuncture Intake Form


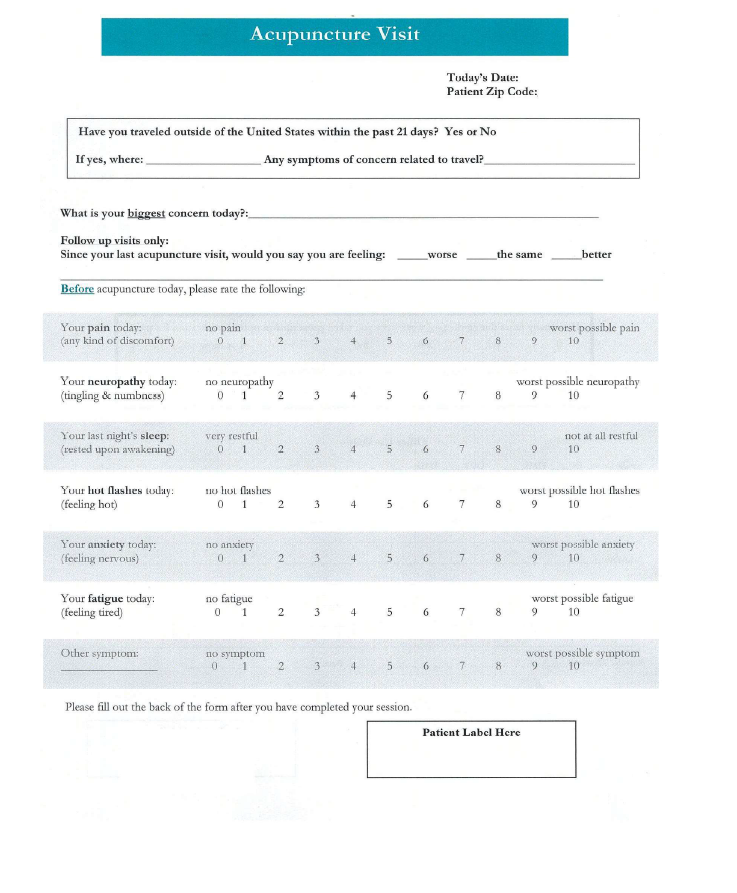


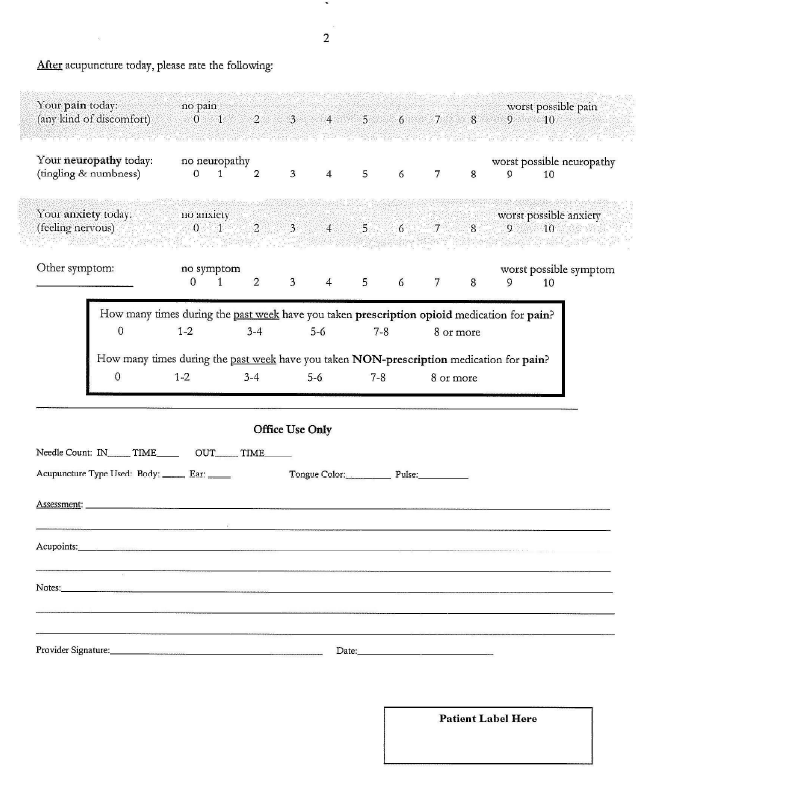

Supplement: Supplementary file 1 — Supplementary file1 (DOCX 563 KB) [file 520_2026_10372_MOESM1_ESM.docx]
