# Supplementary material for: Acupuncture for cancer symptoms: Clinical application and longitudinal impact a retrospective observational real-world data study
Source: Support Care Cancer. 2026 Jan 29;34(2):145. doi: 10.1007/s00520-026-10372-z (PMC12855393; doi:10.1007/s00520-026-10372-z)
Supplement: Supplementary file 2 — Supplementary file2 (DOCX 18 KB) [file 520_2026_10372_MOESM2_ESM.docx]

**Online Resource 2: Patient Characteristics and Acupuncture Utilization by Adherence to Treatment: Single vs Multiple (≥ 2) Acupuncture Sessions**

|  | **One**  **Acupuncture**  **Session**  **N=372** | **Multiple (≥ 2)**  **Acupuncture**  **Session**  **N=1867** | **P-Value** |
| --- | --- | --- | --- |
| **Age** | **Mean (SD)**  **54 (13)** | **Mean (SD)**  **57 (12)** | **P-Value^1^**  **<0.05** |
| **Symptom Burden** | **Mean (SD)**  **2.6 (2)** | **Mean (SD)**  **2.9 (2)** | **P-Value^1^**  **<0.05** |
| **Female Gender** | **Count (%)**  **305 (82%)** | **Count (%)**  **1546 (83%)** | **P-Value^2^**  **NS** |
| **Cancer Diagnosis:**  **Breast**  **Gastrointestinal**  **Gynecological**  **Hematologic**  **Lung**  **Prostate**  **Other** | **Count (%)**  **192 (52%)**  **35 (10%)**  **28 (8%)**  **37 (10%)**  **15 (4%)**  **12 (3%)**  **48 (13%)** | **Count (%)**  **1084 (58%)**  **157 (9%)**  **149 (8%)**  **170 (9%)**  **67 (4%)**  **51 (3%)**  **181 (10%)** | **P-Value^2^**  **NS** |
| **Cancer Treatment (Past/Present)**  **Chemotherapy**  **Surgery**  **Radiotherapy** | **Count (%)**  **238 (64%)**  **246 (66%)**  **160 (43%)** | **Count (%)**  **1141 (61%)**  **1368 (73%)**  **836 (45%)** | **P-Value^2^**  **NS**  **<0.05**  **NS** |
| **Symptom Frequency**  **at Baseline**  **Anxiety (N=827)**  **Fatigue (N=986)**  **Hot Flashes (N=834)**  **Neuropathy (N=927)**  **Pain (N=1355)**  **Sleep Problems (N=1071)** | **Count (%)**  **131 (35%)**  **151 (41%)**  **105 (28%)**  **122 (33%)**  **228 (61%)**  **162 (44%)** | **Count (%)**  **702 (38%)**  **840 (45%)**  **730 (39%)**  **805 (43%)**  **1127 (60%)**  **914 (49%)** | **P-Value^2^**  **NS**  **NS**  **<0.05**  **<0.05**  **NS**  **NS** |
| **Symptom Severity at**  **Baseline**  **Anxiety (N=827)**  **Fatigue (N=986)**  **Hot Flashes (N=834)**  **Neuropathy (N=927)**  **Pain (N=1355)**  **Sleep Problems (N=1071)** | **Median (Q1-Q3)**  **5 (2-7)**  **6 (4-8)**  **6 (4-8)**  **5.5 (3-8)**  **5 (3-7)**  **6 (4-8)** | **Median (Q1-Q3)**  **5 (2-7)**  **5 (3-7)**  **6 (4-8)**  **5 (3-8)**  **5 (3-7)**  **6 (4-8)** | **P-Value^1^**  **NS**  **<0.05**  **NS**  **NS**  **NS**  **NS** |

NS Not Significant; Q1-Q3 interquartile range; SD standard deviation; Symptom Burden is the mean number of symptoms per patient

Number rounded to nearest significant figure except mean symptom burden
