# Supplementary material for: Acupuncture for cancer symptoms: Clinical application and longitudinal impact a retrospective observational real-world data study
Source: Support Care Cancer. 2026 Jan 29;34(2):145. doi: 10.1007/s00520-026-10372-z (PMC12855393; doi:10.1007/s00520-026-10372-z)
Supplement: Supplementary file 3 — Supplementary file3 (DOCX 15 KB) [file 520_2026_10372_MOESM3_ESM.docx]

**Supplement 3. Symptom Severity Before (T1) And Immediately After (A1) The First Acupuncture Session, and the Mean Change (improvement) as Measured on a Severity Numeric Rating Scale (NRS: 0-10)**

|  | **Acupuncture Session** | **N** | **Median Severity NRS (Q1-Q3)** | **Mean Change ± Standard Deviation** | **P-Value*** |
| --- | --- | --- | --- | --- | --- |
| **Anxiety** | **T1** | 827 | 5 (2-7) |  |  |
|  | **A1** | 243 | 2 (0-3) | -3.4 ±2.5 | <0.0001 |
| **Pain** | **T1** | 1346 | 5 (3-7) |  |  |
|  | **A1** | 765 | 2 (0-4) | -2.0 ± 2.1 | <0.0001 |
| **Neuropathy** | **T1** | 919 | 5 (3-8) |  |  |
|  | **A1** | 478 | 4 (2-6) | -1.4 ± 1.9 | <0.0001 |

Q1-Q3 interquartile range

*Wilcoxon Signed-Rank test for pairwise comparison
