# Supplementary material for: Acupuncture for cancer symptoms: Clinical application and longitudinal impact a retrospective observational real-world data study
Source: Support Care Cancer. 2026 Jan 29;34(2):145. doi: 10.1007/s00520-026-10372-z (PMC12855393; doi:10.1007/s00520-026-10372-z)
Supplement: Supplementary file 4 — Supplementary file4 (DOCX 25 KB) [file 520_2026_10372_MOESM4_ESM.docx]

**Brief PubMed Search**

**Search Terms**: “acupuncture” AND “cancer”

**Filter:** “randomized controlled trial”

**Prostate Cancer (2)**

1: Azevedo C, Ferreira da Mata LR, Cristina de Resende Izidoro L, de Castro

Moura C, Bacelar Assis Araújo B, Pereira MG, Machado Chianca TC. Effectiveness

of auricular acupuncture and pelvic floor muscle training in the management of

urinary incontinence following surgical treatment for prostate cancer: A

randomized clinical trial. Eur J Oncol Nurs. 2024 Feb;68:102490. doi:

10.1016/j.ejon.2023.102490. Epub 2023 Dec 12. PMID: 38113770.

2: Frisk J, Spetz AC, Hjertberg H, Petersson B, Hammar M. Two modes of

acupuncture as a treatment for hot flushes in men with prostate cancer--a

prospective multicenter study with long-term follow-up. Eur Urol. 2009

Jan;55(1):156-63. doi: 10.1016/j.eururo.2008.02.002. Epub 2008 Feb 14. PMID:

18294761.

**Breast Cancer (37)**

3: Deng G, Vickers A, Yeung S, D'Andrea GM, Xiao H, Heerdt AS, Sugarman S,

Troso-Sandoval T, Seidman AD, Hudis CA, Cassileth B. Randomized, controlled

trial of acupuncture for the treatment of hot flashes in breast cancer patients.

J Clin Oncol. 2007 Dec 10;25(35):5584-90. doi: 10.1200/JCO.2007.12.0774. Erratum

in: J Clin Oncol. 2008 Mar 20;26(9):1572. D’Andrea, Gabriella M [added]; Xiao,

Han [added]; Heerdt Alexandra S [added]; Sugarman, Stephen [added]; Troso-

Sandoval, Tiffany [added]; Seidman, Andrew D [added]; Hudis, Clifford A [added].

PMID: 18065731.

4: Nedstrand E, Wyon Y, Hammar M, Wijma K. Psychological well-being improves in

women with breast cancer after treatment with applied relaxation or electro-

acupuncture for vasomotor symptom. J Psychosom Obstet Gynaecol. 2006

Dec;27(4):193-9. doi: 10.1080/01674820600724797. PMID: 17225620.

5: Nedstrand E, Wijma K, Wyon Y, Hammar M. Vasomotor symptoms decrease in women

with breast cancer randomized to treatment with applied relaxation or electro-

acupuncture: a preliminary study. Climacteric. 2005 Sep;8(3):243-50. doi:

10.1080/13697130500118050. PMID: 16390756.

6: Crew KD, Capodice JL, Greenlee H, Apollo A, Jacobson JS, Raptis G, Blozie K,

Sierra A, Hershman DL. Pilot study of acupuncture for the treatment of joint

symptoms related to adjuvant aromatase inhibitor therapy in postmenopausal

breast cancer patients. J Cancer Surviv. 2007 Dec;1(4):283-91. doi:

10.1007/s11764-007-0034-x. Epub 2007 Oct 12. PMID: 18648963.

7: Hervik J, Mjåland O. Acupuncture for the treatment of hot flashes in breast

cancer patients, a randomized, controlled trial. Breast Cancer Res Treat. 2009

Jul;116(2):311-6. doi: 10.1007/s10549-008-0210-3. Epub 2008 Oct 7. PMID:

18839306.

8: Walker EM, Rodriguez AI, Kohn B, Ball RM, Pegg J, Pocock JR, Nunez R,

Peterson E, Jakary S, Levine RA. Acupuncture versus venlafaxine for the

management of vasomotor symptoms in patients with hormone receptor-positive

breast cancer: a randomized controlled trial. J Clin Oncol. 2010 Feb

1;28(4):634-40. doi: 10.1200/JCO.2009.23.5150. Epub 2009 Dec 28. PMID: 20038728.

9: Crew KD, Capodice JL, Greenlee H, Brafman L, Fuentes D, Awad D, Yann Tsai W,

Hershman DL. Randomized, blinded, sham-controlled trial of acupuncture for the

management of aromatase inhibitor-associated joint symptoms in women with early-

stage breast cancer. J Clin Oncol. 2010 Mar 1;28(7):1154-60. doi:

10.1200/JCO.2009.23.4708. Epub 2010 Jan 25. PMID: 20100963.

10: Liljegren A, Gunnarsson P, Landgren BM, Robéus N, Johansson H, Rotstein S.

Reducing vasomotor symptoms with acupuncture in breast cancer patients treated

with adjuvant tamoxifen: a randomized controlled trial. Breast Cancer Res Treat.

2012 Oct;135(3):791-8. doi: 10.1007/s10549-010-1283-3. Epub 2010 Dec 14. PMID:

21153699.

11: Frisk J, Källström AC, Wall N, Fredrikson M, Hammar M. Acupuncture improves

health-related quality-of-life (HRQoL) and sleep in women with breast cancer and

hot flushes. Support Care Cancer. 2012 Apr;20(4):715-24. doi:

10.1007/s00520-011-1134-8. Epub 2011 Apr 6. PMID: 21468626.

12: Bokmand S, Flyger H. Acupuncture relieves menopausal discomfort in breast

cancer patients: a prospective, double blinded, randomized study. Breast. 2013

Jun;22(3):320-3. doi: 10.1016/j.breast.2012.07.015. Epub 2012 Aug 18. PMID:

22906948.

13: Molassiotis A, Bardy J, Finnegan-John J, Mackereth P, Ryder DW, Filshie J,

Ream E, Richardson A. Acupuncture for cancer-related fatigue in patients with

breast cancer: a pragmatic randomized controlled trial. J Clin Oncol. 2012 Dec

20;30(36):4470-6. doi: 10.1200/JCO.2012.41.6222. Epub 2012 Oct 29. PMID:

23109700.

14: Smith C, Carmady B, Thornton C, Perz J, Ussher JM. The effect of acupuncture

on post-cancer fatigue and well-being for women recovering from breast cancer: a

pilot randomised controlled trial. Acupunct Med. 2013 Mar;31(1):9-15. doi:

10.1136/acupmed-2012-010228. Epub 2012 Nov 29. PMID: 23196311.

15: Bao T, Cai L, Giles JT, Gould J, Tarpinian K, Betts K, Medeiros M, Jeter S,

Tait N, Chumsri S, Armstrong DK, Tan M, Folkerd E, Dowsett M, Singh H, Tkaczuk

K, Stearns V. A dual-center randomized controlled double blind trial assessing

the effect of acupuncture in reducing musculoskeletal symptoms in breast cancer

patients taking aromatase inhibitors. Breast Cancer Res Treat. 2013

Feb;138(1):167-74. doi: 10.1007/s10549-013-2427-z. Epub 2013 Feb 8. PMID:

23393007; PMCID: PMC3594526.

16: Oh B, Kimble B, Costa DS, Davis E, McLean A, Orme K, Beith J. Acupuncture

for treatment of arthralgia secondary to aromatase inhibitor therapy in women

with early breast cancer: pilot study. Acupunct Med. 2013 Sep;31(3):264-71. doi:

10.1136/acupmed-2012-010309. Epub 2013 May 30. PMID: 23722951.

17: Bao T, Cai L, Snyder C, Betts K, Tarpinian K, Gould J, Jeter S, Medeiros M,

Chumsri S, Bardia A, Tan M, Singh H, Tkaczuk KH, Stearns V. Patient-reported

outcomes in women with breast cancer enrolled in a dual-center, double-blind,

randomized controlled trial assessing the effect of acupuncture in reducing

aromatase inhibitor-induced musculoskeletal symptoms. Cancer. 2014 Feb

1;120(3):381-9. doi: 10.1002/cncr.28352. Epub 2013 Dec 23. PMID: 24375332;

PMCID: PMC3946917.

18: Smith CA, Pirotta M, Kilbreath S. A feasibility study to examine the role of

acupuncture to reduce symptoms of lymphoedema after breast cancer: a randomised

controlled trial. Acupunct Med. 2014 Oct;32(5):387-93. doi:

10.1136/acupmed-2014-010593. Epub 2014 Jul 2. PMID: 24990160.

19: Peter M, Joy B, Jacqueline F, Jennifer FJ, Alexander M. Receiving or not

receiving acupuncture in a trial: the experience of participants recovering from

breast cancer treatment. Complement Ther Clin Pract. 2014 Nov;20(4):291-6. doi:

10.1016/j.ctcp.2014.10.002. Epub 2014 Oct 18. PMID: 25454968.

20: Xiao B, Liu ZH. [Efficacy on depression in breast cancer treated with

acupuncture and auricular acupressure]. Zhongguo Zhen Jiu. 2014

Oct;34(10):956-60. Chinese. PMID: 25543421.

21: Giron PS, Haddad CA, Lopes de Almeida Rizzi SK, Nazário AC, Facina G.

Effectiveness of acupuncture in rehabilitation of physical and functional

disorders of women undergoing breast cancer surgery. Support Care Cancer. 2016

Jun;24(6):2491-6. doi: 10.1007/s00520-015-3054-5. Epub 2015 Dec 15. PMID:

26670916.

22: Greenlee H, Crew KD, Capodice J, Awad D, Buono D, Shi Z, Jeffres A, Wyse S,

Whitman W, Trivedi MS, Kalinsky K, Hershman DL. Randomized sham-controlled pilot

trial of weekly electro-acupuncture for the prevention of taxane-induced

peripheral neuropathy in women with early stage breast cancer. Breast Cancer Res

Treat. 2016 Apr;156(3):453-464. doi: 10.1007/s10549-016-3759-2. Epub 2016 Mar

25. PMID: 27013473; PMCID: PMC4924571.

23: Lesi G, Razzini G, Musti MA, Stivanello E, Petrucci C, Benedetti B, Rondini

E, Ligabue MB, Scaltriti L, Botti A, Artioli F, Mancuso P, Cardini F, Pandolfi

P. Acupuncture As an Integrative Approach for the Treatment of Hot Flashes in

Women With Breast Cancer: A Prospective Multicenter Randomized Controlled Trial

(AcCliMaT). J Clin Oncol. 2016 May 20;34(15):1795-802. doi:

10.1200/JCO.2015.63.2893. Epub 2016 Mar 28. PMID: 27022113.

24: Garland SN, Xie SX, Li Q, Seluzicki C, Basal C, Mao JJ. Comparative

effectiveness of electro-acupuncture versus gabapentin for sleep disturbances in

breast cancer survivors with hot flashes: a randomized trial. Menopause. 2017

May;24(5):517-523. doi: 10.1097/GME.0000000000000779. PMID: 27875389; PMCID:

PMC5403590.

25: Bao T, Iris Zhi W, Vertosick EA, Li QS, DeRito J, Vickers A, Cassileth BR,

Mao JJ, Van Zee KJ. Acupuncture for breast cancer-related lymphedema: a

randomized controlled trial. Breast Cancer Res Treat. 2018 Jul;170(1):77-87.

doi: 10.1007/s10549-018-4743-9. Epub 2018 Mar 8. PMID: 29520533; PMCID:

PMC6159216.

26: Hershman DL, Unger JM, Greenlee H, Capodice JL, Lew DL, Darke AK, Kengla AT,

Melnik MK, Jorgensen CW, Kreisle WH, Minasian LM, Fisch MJ, Henry NL, Crew KD.

Effect of Acupuncture vs Sham Acupuncture or Waitlist Control on Joint Pain

Related to Aromatase Inhibitors Among Women With Early-Stage Breast Cancer: A

Randomized Clinical Trial. JAMA. 2018 Jul 10;320(2):167-176. doi:

10.1001/jama.2018.8907. PMID: 29998338; PMCID: PMC6583520.

27: Brinkhaus B, Kirschbaum B, Stöckigt B, Binting S, Roll S, Carstensen M, Witt

CM. Prophylactic acupuncture treatment during chemotherapy with breast cancer: a

randomized pragmatic trial with a retrospective nested qualitative study. Breast

Cancer Res Treat. 2019 Dec;178(3):617-628. doi: 10.1007/s10549-019-05431-5. Epub

2019 Sep 13. PMID: 31520284.

28: Lu W, Giobbie-Hurder A, Freedman RA, Shin IH, Lin NU, Partridge AH,

Rosenthal DS, Ligibel JA. Acupuncture for Chemotherapy-Induced Peripheral

Neuropathy in Breast Cancer Survivors: A Randomized Controlled Pilot Trial.

Oncologist. 2020 Apr;25(4):310-318. doi: 10.1634/theoncologist.2019-0489. Epub

2019 Oct 14. PMID: 32297442; PMCID: PMC7160396.

29: Zhang ZJ, Man SC, Yam LL, Yiu CY, Leung RC, Qin ZS, Chan KS, Lee VHF, Kwong

A, Yeung WF, So WKW, Ho LM, Dong YY. Electroacupuncture trigeminal nerve

stimulation plus body acupuncture for chemotherapy-induced cognitive impairment

in breast cancer patients: An assessor-participant blinded, randomized

controlled trial. Brain Behav Immun. 2020 Aug;88:88-96. doi:

10.1016/j.bbi.2020.04.035. Epub 2020 Apr 16. PMID: 32305573.

30: Dilaveri CA, Croghan IT, Mallory MJ, Dion LJ, Fischer KM, Schroeder DR,

Martinez-Jorge J, Nguyen MT, Fokken SC, Bauer BA, Wahner-Roedler DL. Massage

Compared with Massage Plus Acupuncture for Breast Cancer Patients Undergoing

Reconstructive Surgery. J Altern Complement Med. 2020 Jul;26(7):602-609. doi:

10.1089/acm.2019.0479. PMID: 32673082; PMCID: PMC7374617.

31: Stöckigt DMB, Kirschbaum B, Carstensen DMM, Witt DMCM, Brinkhaus DMB.

Prophylactic Acupuncture Treatment During Chemotherapy in Patients With Breast

Cancer: Results of a Qualitative Study Nested in a Randomized Pragmatic Trial.

Integr Cancer Ther. 2021 Jan-Dec;20:15347354211058207. doi:

10.1177/15347354211058207. PMID: 34814766; PMCID: PMC8646188.

32: Sicart CSVA, Luz RPC, Rizzi SKLA, Nazário ACP, Facina G, Elias S. Effect of

acupuncture in myelosuppression and quality of life in women with breast cancer

undergoing chemotherapy: a randomized clinical study. Support Care Cancer. 2023

Feb 10;31(3):156. doi: 10.1007/s00520-023-07616-7. PMID: 36763188.

33: Jeong YJ, Choi HR, Kim KS, Shin IH, Park SH. Impact of Acupuncture on Hot

Flashes in Breast Cancer Patients Receiving Adjuvant Antiestrogen Therapy with

Tamoxifen: A Randomized Controlled Trial. J Integr Complement Med. 2023

Apr;29(4):241-252. doi: 10.1089/jicm.2022.0636. Epub 2023 Feb 14. PMID:

36787483.

34: Friedman R, Johnson AR, Shillue K, Fleishman A, Mistretta C, Magrini L, Tran

BNN, Rockson SG, Lu W, Yeh GY, Singhal D. Acupuncture Treatment for Breast

Cancer-Related Lymphedema: A Randomized Pilot Study. Lymphat Res Biol. 2023

Oct;21(5):488-494. doi: 10.1089/lrb.2022.0001. Epub 2023 Apr 20. PMID: 37083501;

PMCID: PMC11708185.

35: Zhang J, Qin Z, So TH, Chang TY, Yang S, Chen H, Yeung WF, Chung KF, Chan

PY, Huang Y, Xu S, Chiang CY, Lao L, Zhang ZJ. Acupuncture for chemotherapy-

associated insomnia in breast cancer patients: an assessor-participant blinded,

randomized, sham-controlled trial. Breast Cancer Res. 2023 Apr 26;25(1):49. doi:

10.1186/s13058-023-01645-0. PMID: 37101228; PMCID: PMC10134666.

36: Bao T, Zhi WI, Baser RE, Li QS, Weitzman M, Gillespie EF, Robson M, Mao JJ.

Electro-acupuncture versus battle field auricular acupuncture in breast cancer

survivors with chronic musculoskeletal pain: subgroup analysis of a randomized

clinical trial. Breast Cancer Res Treat. 2023 Nov;202(2):287-295. doi:

10.1007/s10549-023-07072-1. Epub 2023 Aug 24. PMID: 37612534; PMCID:

PMC11218664.

37: Serra D, Fleishman SB, White C, Leung TM, Chadha M. Acupuncture Reduces

Severity of Hot Flashes in Breast Cancer: A Randomized Single-Blind Trial.

Holist Nurs Pract. 2023 Nov-Dec 01;37(6):330-336. doi:

10.1097/HNP.0000000000000612. PMID: 37851349.

38: Giron PS, Haddad CAS, Lopes De Almeida Rizzi SK, Elias S, Nazário ACP,

Facina G. Effect of Exercise Therapy, Systemic Acupuncture and Silicon Oxide

Tablets on Muscular Strength, Lymphedema, and Quality of Life in Breast Cancer

Survivors: Randomized Clinical Trial. Asian Pac J Cancer Prev. 2025 Mar

1;26(3):799-808. doi: 10.31557/APJCP.2025.26.3.799. PMID: 40156396; PMCID:

PMC12174548.

39: Wang ZN, Tian GH, Ding JR, Lin Y, Zhang LN, Li X, Wei YX, Li XY, Shang QY,

Wang X, Ren L, Du SY, Zhou CT, Wu XL, Lao LX, Tian J, Bai LJ. Acupuncture effect

on brain default mode network connectivity in breast cancer patients with hot

flashes: A randomized controlled trial. Eur J Cancer. 2025 Oct 16;229:115785.

doi: 10.1016/j.ejca.2025.115785. Epub 2025 Sep 12. PMID: 40974865.
